# Biohydrogen Production from Hydrolysates of Selected Tropical Biomass Wastes with *Clostridium Butyricum*

**DOI:** 10.1038/srep27205

**Published:** 2016-06-02

**Authors:** Dan Jiang, Zhen Fang, Siew-xian Chin, Xiao-fei Tian, Tong-chao Su

**Affiliations:** 1 University of Science and Technology of China, School of Life Science, 443 Huangshan Road, Hefei, Anhui Province 230022, China; 2 Chinese Academy of Sciences, Biomass Group, Key Laboratory of Tropical Plant Resources and Sustainable Use, Xishuangbanna Tropical Botanical Garden, 88 Xuefulu, Kunming, Yunnan Province 650223, China; 3 Biomas Group, College of Engineering, Nanjing Agricultural University, 40 Dianjiangtai Road, Nanjing, Jiangsu 210031, China; 4 Department of Chemical and Process Engineering, Faculty of Engineering and Built Environment, Universiti Kebangsaan Malaysia, 43600 UKM, Bangi, Selangor, Malaysia; 5 School of Bioscience and Bioengineering, South China University of Technology, 382 Outer ring east road, Higher Education Mega Centre, Guangzhou, 510006, China

## Abstract

Biohydrogen production has received widespread attention from researchers in industry and academic fields. Response surface methodology (RSM) was applied to evaluate the effects of several key variables in anaerobic fermentation of glucose with *Clostridium butyrium*, and achieved the highest production rate and yield of hydrogen. Highest H_2_ yield of 2.02 mol H_2_/mol-glucose was achieved from 24 h bottle fermentation of glucose at 35 °C, while the composition of medium was (g/L): 15.66 glucose, 6.04 yeast extract, 4 tryptone, 3 K_2_HPO_4_, 3 KH_2_PO_4_, 0.05 L-cysteine, 0.05 MgSO_4_·7H_2_O, 0.1 MnSO_4_·H_2_O and 0.3 FeSO_4_·7H_2_O, which was very different from that for cell growth. Sugarcane bagasse and *Jatropha* hulls were selected as typical tropical biomass wastes to produce sugars *via* a two-step acid hydrolysis for hydrogen production. Under the optimized fermentation conditions, H_2_ yield (mol H_2_/mol-total reducing sugar) was 2.15 for glucose, 2.06 for bagasse hydrolysate and 1.95 for *Jatropha* hull hydrolysate in a 3L fermenter for 24 h at 35 °C, with H_2_ purity of 49.7–64.34%. The results provide useful information and basic data for practical use of tropical plant wastes to produce hydrogen.

Hydrogen is a clean fuel that only produces water as its environmentally benign product in combustion, and therefore attracts attentions from researchers in the world^1^. The specific energy of hydrogen (122 kJ/g) is 2.75 times that of regular hydrocarbon fuels^2^. Annual global production of lignocellulosic biomass is about 2.20 × 10^12^ Kg (dry weight) from agriculture and forestry residuals, energy crops, aquatic plants and algae^3^. These rich and sustainable lignocellulosic resources potentially produced H_2_ with low cost from biomass *via* hydrolysis and fermentation^4^.

South China is located in subtropical and tropical regions that were suitable to grow energy or economic crops, such as sugarcane and *Jatropha curcas* L^5^. In China, sugarcane production increased from 21.12 in 1978 to 128.2 million tons in 2013, which produced large amount of bagasse as waste in sugar industry^6^. Large amount of bagasse was produced as industrial waste, which could be used to product biofuels such as bioethanol and biohydrogen^7^,^8^. On the other hand, *Jatropha* is a promising energy crop for biodiesel industry. More than 2,000 biodiesel production plants had been built up in China by 2007 with many residual hulls produced^9^,^10^. *Jatropha* hulls were previously studied to produce 2.3-butanediol and biogas^11^,^12^.

Lignocellulosic biomass has complex structure composing of cellulose, hemicellulose and lignin, which should be disrupted before hydrolyzing cellulose and hemicellulose to soluble sugars^13^. Various types of agricultural residues (such as rice straw and corn stover) were hydrolyzed with dilute-acid for the production of hydrogen^14^,^15^. Compared with hydrochloric acid, nitric acid and phosphoric acid, sulfuric acid was more effective in producing fermentable sugars^16^. Most hemicellulose was hydrolyzed in the first step. The second step was then performed at severer conditions to open up the cellulose structure for improving the accessibility of enzymes for hydrolysis, or to hydrolyze cellulose with acid^17^. Therefore, two-step dilute acid hydrolysis process was one of the effective pretreatment methods.

*Clostridium butyricum* as a model bacterium with clear metabolic pathways, was widely used in hydrogen production^18^,^19^,^20^. Preliminary results suggested that the optimized fermentation medium by response surface methodology (RSM) improved the growth of *C. butyricum* effectively^21^. However, the most optimal culture medium for the strain growth was not optimal for H_2_ yield. To the best of our knowledge, the hydrolysate of *Jatropha* hulls for biohydrogen production has not yet been reported in any previous work. This study aims to (i) optimize the fermentation media for biohydrogen production using glucose as carbon source by RSM, and (ii) the results was further used to efficiently produce biohydrogen from the hydrolysates of bagasse and *Jatropha* hulls by two-step acid hydrolysis.

## Results and Discussion

**Figure 1:**
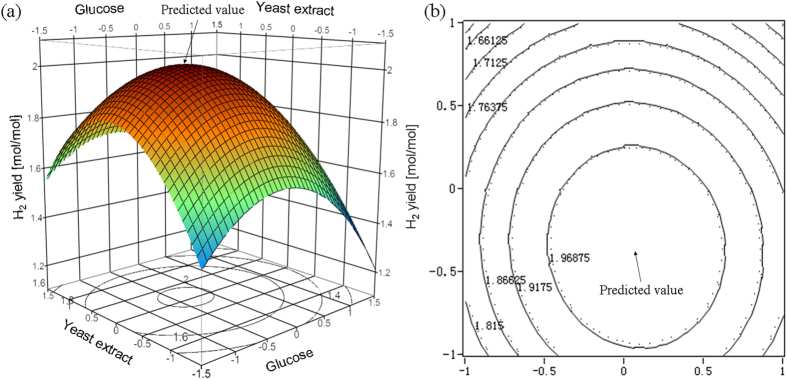
(**a**) Response surface plot and (**b**) corresponding contour of the mutual effects of glucose and yeast extract on H_2_ yield (24 h bottle fermentation at 35 °C with 130 rpm shaking).

**Figure 2:**
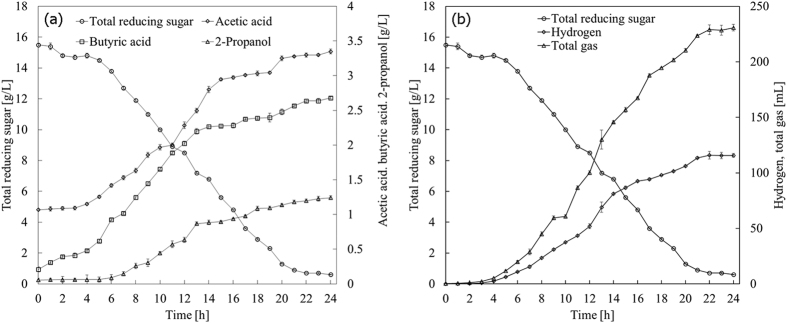
H_2_ and by-products production versus time in bottle fermentation of bagasse hydrolysates under the optimized medium at 35 °C with 130 rpm shaking (**a**) liquid products (**b**) gas products.

**Figure 3:**
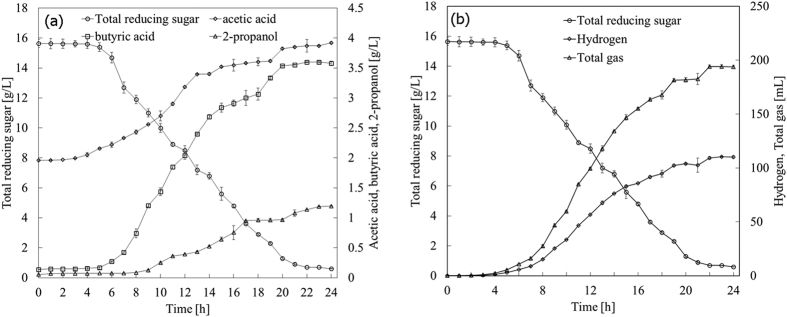
H_2_ and by-products production versus time in bottle fermentation of *Jatropha* hulls hydrolysates under the optimized medium at 35 °C with 130 rpm shaking (**a**) liquid products (**b**) gas products.

Glucose and hydrolysates were fermented with *C. butyrium* in both bottles (batch) and a fermenter. Single-factor bottle experiments were conducted to find best culture medium for hydrogen production from glucose with results given in supplementary materials (Figure S1). Linear relation between dry cell weight (DCW) and optical density at 650 nm (OD_650_) was plotted in supplementary materials (Figure S2). Both H_2_ yield and DCW versus different variables were illustrated in supplementary materials (Figure S3). Plackett-Burman (PB) design, experimental results and analysis with the help of software JMP package (version 10, SAS Institute Inc., Raleigh, NC) were demonstrated in Tables 1, 2, 3, [Table t3], respectively. Study on the path of steepest ascent could be observed in Table 4. Central composite design (CCD), experimental results and analysis were summarized in Tables 5 and 6. Response surface plot and corresponding contour, canonical analysis for RSM were given in Fig. 1 and Table 7, respectively. Products versus time in bottle fermentation of hydrolysates of bagasse and *Jatropha* hulls were showed in Figs 2 and 3. Composition analysis of bagasse and *Jatropha* hulls was given in Table 8. Products produced from glucose and hydrolysates in both bottles and fermenter were presented in Table 9. Table 10 Compares hydrogen yield in this study with reported data. All the experiments were repeated more than two times and the standard deviation (σ) was within the range of 0.002 to 0.11 (mol/mol) for H_2_ production, and 0.008 to 0.09 (g/L) for microbial growth. Detailed results were presented and discussed below.

### Single-factor experiments

Single-factor experiments were performed to verify factors in fermentation medium that affected H_2_ yield, and to provide basic data for following experiments. Batch (bottle) fermentation was performed at 35 °C for 24 h with the culture medium conditions given in Method section and shaking rate of 130 rpm. It can be concluded from Figure S1 that fermentation medium producing the highest H_2_ yield of 1.67 mol H_2_/mol-glucose contained (g/L): 15 glucose, 5 yeast extract, 3 K_2_HPO_4_, 3 KH_2_PO_4_, 0.05 L-cysteine, 0.05 MgSO_4_·7H_2_O, 0.1 MnSO_4_·H_2_O and 0.3 FeSO_4_·7H_2_O, as compared to the yield of 1.28 mol H_2_/mol-glucose (increased by 30.47%) with the initial medium. Microbial growth was also studied under different variable concentrations in culture medium. Regression curve presents well-linear relationship between DCW and OD_650_ (Figure S2). It was found that the optimal concentrations of variables for microbial growth and H_2_ yield were different (Figure S3). Obviously, the optimal concentrations of glucose, L-cysteine, MgSO_4_·7H_2_O and FeSO_4_·7H_2_O were 30, 0.1, 0.15 and 0.1 g/L for DCW, respectively, while they were 15, 0.05, 0.05 and 0.3 g/L for H_2_ production (Figure S3). The most optimal culture medium for the strain growth was not the maximum for hydrogen production.

### PB design

PB design was used to identify variables that had significant effects on H_2_ production. The medium compositions were: glucose, yeast extract, tryptone, K_2_HPO_4_, KH_2_PO_4_, L-cysteine, MgSO_4_·7H_2_O and FeSO_4_·7H_2_O (Table 1). Both high and low levels (**1** and **−1**) of each variable were chosen based on the above preliminary single-factor experiments. Table 2 gave the PB experiment results in 24 h batch fermentation at 35 °C with 130 rpm, while the statistical analysis of PB experiment data was summarized in Table 3. The significance of each variable was determined by their corresponding *p*-values. Factors evidencing *p*-values of less than 0.05 were considered to be significant effects on the response^22^, and were subsequently studied in further optimization studies. In this case, X_1_ (glucose; *p* = 0.0015) and X_2_ (yeast extract; *p* = 0.0181) were statistically significant in the production of H_2_ (*p* < 0.05). X_1_ had the greatest negative impact on H_2_ yield. On the other side, X_2_ had positive effect on H_2_ yield. X_3_ (tryptone), X_5_ (KH_2_PO_4_), X_7_ (MgSO_4_·7H_2_O) and X_8_ (FeSO_4_·7H_2_O) were set at their high levels according to the positive effects although they were insignificant to H_2_ yield. Factors such as X_4_ (K_2_HPO_4_) and X_6_ (L-cysteine) with negative effects were selected on their low levels. To approach the optimum response, a fitted first-order model equation for H_2_ yield (Y) was obtained from the PB design experiments:


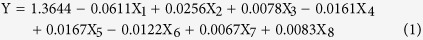


The coefficient of each variable in Eq. (1) represents the effect weight of the variable on H_2_ yield. The quality of the fitted polynomial model equation was expressed by the coefficient of determination (R^2^). The obtained R^2^ was 0.9858, indicating that 98.58% variability in the response could be explained by the model, but only 1.42% variability was not explained. Meanwhile, the value of adjusted determination coefficient (Adj R^2^ = 0.9478) was very high, which advocates a high significance of the model^23^. These results show that the response equation provides a suitable model for the PB design experiments.

### Path of the steepest ascent

Experiments were started from zero level in PB design. If *t*-value was positive, climbing direction would increase and vice versa^24^. Overall, step size was determined by the above *t*-values (Table 3) and single-factor experiments. According to *t*-values (Table 3) and single-factor results, step size of 2 and 0.5 g/L was set for glucose and yeast extract, respectively. Glucose shows decreasing trend but an increasing trend for yeast extract. The concentrations of other factors were steadfast in all trials at their optimal levels. It was observed that H_2_ yield increased along the path from test 1 to 3, reached the peak of 1.78, and decreased from test 4 to 7. This means that the optimal level was close to that in test 3 (16.0 g/L glucose and 6.0 g/L yeast extract). In Table 4, the highest H_2_ yield was 1.78 mol H_2_/mol-glucose for test 3. In these experiments, H_2_ yield was remarkably improved. This suggests that the steepest ascent method was an effective technique to determine an optimal level. However, the optimal values for these two variables need to be determined *via* the following CCD.

### RSM and CCD

A series of experiments were carried out *via* RSM to obtain an optimal combination of glucose and yeast extract. Table 5 gave five different coding levels, full experimental design and the results of CCD, with the center point set based on the results in steepest ascent test (Table 4).

Table 6 summarizes the significance test of regression coefficients. Using multivariate regression analysis of the observed data (Table 6), the obtained model illustrates the relationship between glucose (X_1_) and yeast extract (X_2_) corresponding to H_2_ yield (Y) as below:


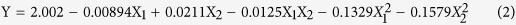


where Y was the predicted H_2_ yield, X_1_ and X_2_ were the coded values of glucose and yeast extract. The regression model (Eq. 2) can be used to predict the range of H_2_ production for various levels of the selected variables.

Determination and adjusted determination coefficients (R^2^ and Adj R^2^) were calculated as 91.72% and 85.8% for the regression, demonstrating the agreement between the experimental and predicted H_2_ yields that provides a good estimation of the response within the range of process conditions.

Analysis of variance (ANOVA) is essential to test the significance and adequacy of model. High *t*-value reveals an adequate explanation of the variation of data regarding to their mean value. The *t*-test and *p*-values served to examine the significance of each coefficient, which also illustrated the interaction strength among independent variables^25^. The *t*-test of each significant variable level was given in Table 6. In this model term, the linear and quadratic of X_1_, X_1_X_1_ and X_2_X_2_ (*p* < 0.05) were recognized as the significant factors^26^, suggesting that H_2_ yield was directly related to these two main factors (glucose and yeast extract). However, the interaction between glucose and yeast extract (X_1_X_2_, *p* > 0.05) seemed to be less significant on H_2_ production. *F*-values of the model and lack of fit were 170.16 and 2.03, respectively (Table 7), while model *p*-values and *p*-values of lack of fit were <0.0001 and 0.2528, suggesting that the model was good fit to the experimental data.

Three dimensional (3D) response surface and corresponding contour of glucose and yeast extract were presented in Fig. 1. The response surface (Fig. 1a) displays an obvious convex, revealing the optimum conditions were well-defined. The contour plot was almost circular, suggesting that the interaction of glucose and yeast extract had a less significant effect on H_2_ production. In the surface plot, H_2_ yield achieved the highest value within the studied region. Canonical correlation analysis (CCA) is a multivariate technique focused on determining the relationship among groups of variables in a datum set^27^. CCA was further conducted (Table 7), at X_1_ = −0.3402 (glucose, 15.66 g/L) and X_2_ = 0.0804 (yeast extract, 6.04 g/L), the predicted maximum H_2_ yield corresponding to these values was 2.02 mol H_2_/mol-glucose.

### Experimental validation of the optimized medium

In order to verify the model (Eq. 2) adequacy in predicting the maximum yield of H_2_, experiments were performed under optimum medium composition with 3 repetitions. H_2_ yield was 2.03 ± 0.02 mol H_2_/mol-glucose after 24 h bottle fermentation that agrees excellently with the predicted value (2.02 mol H_2_/mol-glucose).

In summary, RSM is an efficient tool to optimize the medium composition for hydrogen production by *C. butyrium.* The optimized medium was (g/L): 15.66 glucose, 6.04 yeast extract, 4 tryptone, 3 K_2_HPO_4_, 3 KH_2_PO_4_, 0.05 L-cysteine, 0.05 MgSO_4_·7H_2_O, 0.1 MnSO_4_·H_2_O and 0.3 FeSO_4_·7H_2_O with 2.03 mol H_2_/mol-glucose after 24 h. On the other hand, previous study gives the optimum fermentation medium for the growth of *C. butyricum* was composed of (w/v): 2% glucose, 0.5% pectin, 0.2% casein, 3.98% soyabean cake extract, 0.1% (NH_4_)_2_SO_4_, 0.124% NaHCO_3_, 0.37% corn steep flour, 0.02% MnSO_4_·H_2_O, 0.02% MgSO_4_·7H_2_O and 0.002% CaCl_2_ at *pH* 7.5^21^. The optimized media for growth and H_2_ production were different. All the above experiments were conducted in sealed 100 mL bottles. The obtained optimized results were further used for the production of H_2_ from actual biomass wastes below.

### Hydrogen from hydrolysates of bagasse and *Jatropha* hulls

Bagasse and *Jatropha* hulls mainly consist of cellulose and hemicellulose and lignin^11^,^18^. The chemical compositions of bagasse and *Jatropha* hulls were analyzed according to the technical report from US National Renewable Energy Laboratory (NREL)^28^,^29^ (Table 8). Bagasse consisted of 25.17 wt% hemicellulose and 42.05 wt% cellulose, but 13.37 wt% hemicellulose and 36.95 wt% cellulose for *Jatropha* hulls. Bagasse and *Jatropha* hulls were hydrolyzed by two-step dilute acid hydrolysis in an autoclave. After reaction, the hydrolysates were neutralized, concentrated, detoxificated, and fermented under the optimized medium obtained from glucose. Figures 2 and 3 illustrated liquid and gas products *vs.* time in the bottle fermentation of hydrolysates from bagasse and *Jatropha* hulls, respectively. For the fermentation of bagasse hydrolysate (Fig. 2), as time increased, yields of H_2_, acetic acid and butyric acid increased with the decreasing TRS. These liquid products rose very slowly before 4 h as the strain was in the lag phase for a short adaptation to the new environment^30^. In Fig. 2a, after 5 h, acetic acid and butyric acid grew rapidly to 3.25 and 2.48 g/L at 20 h (*vs.* 1.26 and 0.62 g/L at 5 h), and slightly rose further to 3.35 and 2.68 g/L at 24 h, respectively. Acetate and butyrate rose slightly after 15 h and stopped increasing after 20 h with the consumption of TRS because the accumulation of acetate and butyrate caused a sharp drop in culture pH and inhibited the growth of bacteria. 2-Propanol also presented similar rising trend. Similarly, H_2_ production (Fig. 2b) rose sharply from 3.05 to 115.87 mL at time from 4 to 22 h, and changed little until 24 h. At the same time, total gas rose much higher from 5.29 at 4 h to 228.9 mL at 22 h because CO_2_ was produced. For the fermentation of hydrolysate of *Jatropha* hulls (Fig. 3a), acetic acid and butyric acid increased rapidly to 3.87 and 3.59 g/L at 22 h from 2.16 and 0.17 g/L at 5 h. However, more butyric acid (3.52 g/L) produced at 24 h from the hydrolysate of *Jatropha* hulls than that of bagasse (2.68 g/L). The initial acetic acid concentration from the hydrolysate *of Jatropha* hulls (1.96 g/L) was higher than that of bagasse (1.06 g/L) that may inhibit the further production of acetic acid. More acetic acid was produced from the hydrolysate of bagasse (2.28 g/L) than *Jatropha* hulls (1.97 g/L) at 24 h (Table 9). Contrary, the production of butyric acid from the hydrolysate *Jatropha* hulls (3.43 g/L) was more than that of bagasse (2.47 g/L). The growth of strains was likely to be inhibited by the acetate produced when grown on media containing glucose^31^. So, less H_2_ was produced from hydrolysate of *Jatropha* hulls (110.4 *vs.* 115.87 mL for bagasse hydrolysate at 24 h) *via* acetic route discussed below.

Sugars were also fermented in a 3L fermenter for 24 h at 35 °C with 130 rpm stirring (Table 9). H_2_ yield from glucose is 2.15 (mol H_2_/mol-glucose), which is slightly higher than the experiment in bottle fermentation (2.02 mol H_2_/mol-glucose) because hydrogen pressure in bottle fermentation increased as gas accumulated in the fixed 100-mL bottle that may prevent hydrogen production. But, no pressure rose for the fermenter since it was connected on-line to an empty gas bag. Previous study shown that partial pressure of H_2_ (pH_2_) was an extremely important factor for continuous H_2_ synthesis^32^. The hydrogen evolution rate and yield were improved by 10% and 15%, respectively, by reducing the H_2_ partial pressure through pumping out produced gas in a fermentor with silicone rubber^33^. So, high pressure prevented hydrogen production in bottle fermentation. The hydrogen production also affected by its concentration. As hydrogen concentration increased, H_2_ synthesis decreased and metabolic pathways shifted towards the production of more reduced substrates, such as lactate, ethanol, acetone, butanol or alanine^32^. On the other hand, acids produced in the bottles without neutralization may also hamper the production of H_2_. Acetate accumulation caused a sharp drop of culture pH and subsequent inhibition of bacterial hydrogen production, that were reported previously^34^. For fermenter experiments, H_2_ yield from hydrolysates of bagasse and *Jatropha* hulls is 2.06 and 1.95 (mol H_2_/mol-TRS), which reached 95.8% and 90.7% of that from pure glucose, respectively.

Generally, glucose or other reducing sugars (e.g., xylose) were the preferred carbon source for fermentation with overall reactions as below^35^:


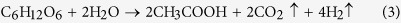



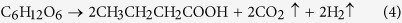


According to Eq. (3), 4 mol H_2_/mol-glucose was obtained with acetic acid and CO_2_ as by-products. However, if butyric acid was formed as by-product, only 2 mol H_2_/mol-glucose was produced (Eq. 4). The ratio of acetic/butyric acids obtained could be related to the production of H_2_. Previous studies showed that the increase of the ratio was accompanied by increased production of H_2_^35^. In Table 9, it was well-confirmed in fermenter that acetic/butyric acid ratio was 0.67, 1.03 and 1.15 (g/g) for *Jatropha* hulls, bagasse and glucose, corresponding to their H_2_ yield (mol/mol-TRS) of 1.95, 2.06 and 2.15, respectively. As comparison, bottle experiments with lower acetic/butyric acid ratio (g/g) of 0.57, 0.9 and 1.09 for hydrolysates of *Jatropha* hulls, bagasse and glucose had lower H_2_ yield (mol/mol-TRS) of 1.89, 1.99 and 2.03 (Table 9). Gas is composed of 49.7–64.34% H_2_ and 35.66–50.29% CO_2_ from anaerobic fermentation in bottles and fermenter. H_2_ from glucose (64.34%) had much higher concentration than those from hydrolysates of bagasse (53.92%) and *Jatropha* hulls (49.7%) in fermenter. The concentrations of H_2_ produced from glucose and bagasse in fermenters were higher than those in bottles (64.34% and 53.92% *vs.* 52.69% and 50.16%). However, H_2_ produced from *Jatropha* hulls (49.7%) in fermenter was less pure than that in bottles (56.62%).

Table 10 compares biohydrogen production in this work with other previous studies from various types of biomass with different microorganisms. In those previous studies, the yield of H_2_ fermented by bagasse hydrolysate was 0.8–1.73 mol H_2_/mol-TRS^18^,^19^, and 0.85–2.3 mol H_2_/mol-glucose^36^,^37^,^38^. This study produced comparable biohydrogen yields to the reported studies (1.95–2.15 *vs.* 0.8–2.3 mol H_2_/mol-TRS).

## Conclusions

Fermentation medium for H_2_ production from glucose with *Clostridium butyricum* was optimized by response surface methodology with the highest H_2_ yield of 2.02 mol H_2_/mol-glucose for batch fermentation at 35 °C for 24 h. The optimized medium was composed of (g/L): 15.66 glucose, 6.04 yeast extract, 4 tryptone, 3 K_2_HPO_4_, 3 KH_2_PO_4_, 0.05 L-cysteine, 0.05 MgSO_4_·7H_2_O, 0.3 FeSO_4_·7H_2_O and 0.1 MnSO_4_·H_2_O. Higher H_2_ yield of 2.15 mol H_2_/mol-glucose was achieved for 24 h in a well-controlled fermenter. The hydrolysates of bagasse and *Jatropha* hulls from two-step dilute acid hydrolysis were further successfully fermented to hydrogen with yields of 2.06 and 1.95 (mol H_2_/mol-total reducing sugars) in fermenter, respectively. Higher H_2_ yield from glucose and bagasse hydrolysate was due to their high acetic/butyric acid ratio *via* acetic route for hydrogen production. It is also found that optimized condition for H_2_ production was not the best condition for microbial growth. Tropical biomass wastes as inexpensive raw materials can be effectively produce biohydrogen.

## Methods

### Materials

Substrates [glucose (99.8%), xylose (99.9%), fructose (99.5%), galactose (99.8%) and mannose (99.7%)], yeast extract, L-cysteine and tryptone were bought from Bomei Biotech Co., Ltd. (Heifei, Anhui). Calcium carbonate (99.8%), K_2_HPO_4_ (99%), KH_2_PO_4_ (99.8%), Na_2_HPO_4_ (99%), NaCl (99.5%), MnSO_4_·H_2_O (99%), MgSO_4_·7H_2_O (99%, FeSO_4_·7H_2_O (99%), glycerol (99%), ethanol (99.7%) and sulfuric acid (98%) were bought from Xilong Chemical Factory Co., Ltd. (Shantou, Guangdong). Standard sugars of glucose, xylose, arabinose, mannose and galactose (purity > 99%) were bought from Sigma-Aldrich (Shanghai). Acetic acid (99.5%), butyric acid (99.7%) and 2-propanol (99.9%) were from Aladdin Industrial Corporation (Shanghai). Activated carbon (powder, 97%) was purchased from Fengchuan Chemical Reagent Co., Ltd (Tianjin).

*Jatropha* hulls were purchased from Yunnan Shenyu New Energy Co., Ltd. (Chuxiong, Yunnan). Bagasse was bought from Dehong (Yunnan). Biomass samples were dried at 45 °C until constant weight (WFO-710, EYELA, Tokyo Rikakikai Co., Ltd.), ground in a pulverizer (9FC-15, Xudong Machinery Manufacturing Co., Ltd., Leshan, Sichuan) and sieved through 80 mesh for analysis and hydrolysis.

### Microorganism and batch (bottle) fermentation

*C. butyricum* (CICC 20763) used for producing H_2_ was purchased from China Center of Industrial Culture Collection (CICC, Beijing). The culture was maintained on corn meal agar stab at 4 °C. The seed medium composed of (g/L): 20 glucose, 5 yeast extract, 5 tryptone and 3 NaCl with *pH* 6.5. To make the seed cultivation, a loop of *C. butyricum* from a fresh slant tube was inoculated into a serum bottle (250 mL) containing 50 mL seed medium and then incubated in a rotary shaker (ZWY-2102C, Zhicheng Analytical Instrument Manufacturing Co., Ltd., Shanghai) at 35 °C with 130 rpm for 12 h.

The initial medium for anaerobic fermentation was composed of (g/L): 25 glucose, 10 tryptone, 5 Na_2_HPO_4_, 3 NaCl and 0.05 FeSO_4_·7H_2_O. A 4% (v/v) of the seed culture was inoculated into the medium for fermentation. After sterilization at 121 °C for 20 min in an autoclave (HVE-50, Hirayama Manufacturing Corp., Tokyo), substrates with *C. butyricum* (about 20 mL, initial *pH* 6.5) were loaded into 100-mL bottles in an anaerobic incubator (YQX-II, CIMO Medical Instrument Manufacturing Co., Ltd., Shanghai) to maintain anaerobic environment by nitrogen purge (99.999% N_2_, Meisel Gas Products Co., Ltd., Kunming, Yunnan) and sealed by rubber-aluminium cap. The bottles with samples were put in an incubator shaker (ZWY-2102C) at 130 rpm and 35 °C for batch fermentation. After fermentation (up to 24 h), gas was collected in a bag (500 mL) by a 50 mL syringe (Agilent Inc., Palo Alto, CA) and its volume (e.g., up to 233 mL at 24 h) was measured by the same syringe. Gas was analyzed by gas chromatograph (GC) and liquid sample by high performance liquid chromatograph (HPLC), ultraviolet (UV)-visible spectrophotometer and biosensor introduced in detail below.

### PB design

PB design was applied to select factors that significantly influenced H_2_ yield, based on the first-order (linear) model as below^22^:


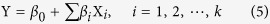


where Y was H_2_ yield as response; X_i_ was coded independent factor and *β*_i_ was linear coefficient; *β*_0_ was intercept value. Each variable had two-levels, high and low, coded by (**+1**) and (**−1**), respectively.

### Path of steepest ascent method

The method of steepest ascent given by Box and Wilson is a procedure for moving sequentially along the direction of the maximum increase in response^39^. The direction in which H_2_ yield increased most rapidly was that of steepest ascent, started from the zero level of variables (significant factors) in PB design, while the step size was decided by the estimated coefficient ratio from Eq. 5, together with practical experience. This test was disused until response no longer increased. As a result, the steepest ascent method allowed factors approaching the optimal level and gave a more limited region for RSM optimization^40^.

### CCD and RSM

CCD was employed to optimize the two most significant factors (glucose and yeast extract) for the maximum H_2_ yield, screened by PB design. These two independent factors were studied at five different levels (**−1.41, −1, 0, +1** and **+1.41**), and a set of 13 experiments were carried out (Table 5). The behavior of the experiment was explained by the following second-order polynomial equation^23^:


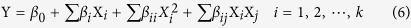


where Y was the predict response, *β*_0_ was intercept, X_i_ and X_j_ were input variables which influenced the response Y, *β*_i_ was linear coefficient, *β*_ii_ was quadratic coefficient, and *β*_ij_ was interaction coefficient. The second-order polynomial coefficients were also calculated.

### Composition analysis of bagasse and *Jatropha* hulls

Composition analysis of bagasse and *Jatropha* hulls was conducted according to the standard US NREL protocols^28^,^29^. All the raw materials were dried at 45 °C until achieving constant weight. Biomass sample (2–10 g, oven dry weight, ODW) was extracted in a Soxhlet extractor with deionized water and ethanol at boiling points for 12–24 h, respectively. The extracted solutions were dried in a flask to remove water and ethanol at 45 to 60 °C by a rotary evaporator. The extracted solid biomass (0.3 ± 0.01g, dried) was hydrolyzed in a 25-mL tube containing 4.98 ± 0.01 g concentrated sulfuric acid (72 wt%) stirred by a magnetic bar at 30 °C water bath for 1 h, and then diluted to 4 wt% sulfuric acid solution by adding 84 ± 0.01 mL deionized water. The diluted solution with biomass sample was put in a 100 mL bottle sealed by a rubber-aluminum cap and autoclaved at 121 °C for 60 ± 1min (HVE-50) for second-step hydrolysis, at the same time, a reference standard sugar mixture (glucose, xylose, galactose, arabinose and mannose) with similar concentrations to the sugars in biomass sample was also put in the autoclave to calibrate the decomposition of each sugar. The filtrated supernatant was collected for acid-soluble lignin determination by using UV-Visible spectrophotometer at 240 nm wavelength (UV-1800, Shimadzu, Kyoto). The supernatant was neutralized to *pH* 5.0–6.0 with calcium carbonate for sugar analysis described below. The solid residual separated from the hydrolysate by filtration (0.22 μm pore size) was oven-dried for acid-insoluble lignin determination. Crucibles containing residues were placed in a muffle furnace (4–10, Ever light medical equipment Co., Ltd, Beijing) at 575 ± 25 °C for 24 h to determine ash weight.

### Hydrolysis of *Jatropha* hulls and bagasse

Two-step dilute acid hydrolysis was conducted in a 500-mL high-pressure autoclave (FCFD05-30, Yantai Jianbang Chemical Mechanical Co. Ltd., Shandong). In the first-step hydrolysis, bagasse or *Jatropha* hulls (20 g, dry weight) mixed with 2 wt% sulphuric acid solution with solid-liquid ratio (SLR) of 1:10 (g/g) was hydrolyzed at 130 °C for 1 h^41^. After reaction, solid residue was filtrated (0.22 μm pore size), washed and dried for second-step hydrolysis in 4 wt% sulphuric acid solution with 15 g solid residue, SLR of 1:10 (g/g) at 150 °C for 1 h^11^. Hydrolysates from both steps were mixed together, and neutralized to *pH* 6.0 with calcium hydroxide. After filtration (0.22 μm pore size), the solution was concentrated to 15.66 g/L TRS by a rotary evaporator at 60–70 °C. The concentrated solutions (100 mL) were further detoxicated with 2 wt% activated carbon at 50 °C for 2 h, and used for the following fermentations. The hydrolysates were fermented under the optimized medium obtained from glucose in bottles because they mainly contained hexose and pentose sugars with minor of acids, furfural, hybrid protein and other toxic components that were neutralized and detoxified.

### Fermentation in a fermenter

Fermentation of glucose and hydrolysates (about 1 L) with *C. butyricum* was also carried out in a 3L fermenter (LiFlus GX, Hanil Science Industrial Co. Ltd., Incheon, South Korea) at 35 °C for 24 h with 130 rpm stirring. Its pH was controlled at 6.5 by adding 1 M sodium hydroxide or 1 *M* hydrochloric acid. Gas generated (up to 8.8 L) during the fermentation process was collected into a gas bag (10 L) on-line and its volume was measured after fermentation using a wet gas meter (LMF-1, Shanghai A.K. Instruments Co., Ltd.), where water was saturated by NaHCO_3_ to avoid absorption of CO_2_. Gas and liquid products were analyzed described below.

### Analytical methods

Gas was analyzed by GC (7820A, Agilent, Palo Alto, CA) equipped with four columns [three packed columns: one Porapak Q (9 ft × 1/8 in.) and two Porapak N (3 ft × 1/8 in.), and one Molecular Sieve 5A (6 ft × 1/8 in.)] with two thermal conductivity detectors (TCD). In this work, no CH_4_, CO, C_2_H_6_ and C_2_H_2_ gases were detected. Produced gases (H_2_ and CO_2_) were only separated by Porapak Q (CH_4_ together with CO were also monitored even though their peaks were overlapped). Helium (99.999% purity, Guangruida Co. Ltd., Kunming, Yunnan) was used as carrier gas. Gas sample was calibrated with a standard gas mixture (35% H_2_, 2% CH_4_, 25% CO_2_; v/v) (Huate Gas Co. Ltd., Foshan, Guangdong), and diluted 1–5 times into five gradients with nitrogen (99.9999%, Guangruida Co. Ltd.). All the standard calibration curves obtained had R^2^ > 0.999. H_2_ yield was defined as below:


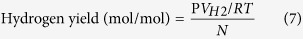


where P (0.8 × 10^5^ Pa in Kunming at an altitude of 1,896 m, calculated by Barometric formula^42^) is atmospheric pressure; V_H2_ is gas volume (m^3^); R is perfect gas constant (8.3144, m^3^·Pa·mol^−1^ · K^−1^); T is gas temperature (298 K), and N is mole number of substrate (e.g., glucose, TRS, glycerol] before fermentation (mol).

Liquid samples (glucose, xylose, acetic acid and butyric acid) after fermentation were measured by HPLC (LC-20A, Shimadzu) equipped with a refractive index detector (RID, Shimadzu) setting at 55 °C and Aminex HPX-87H column (Bio-Rad, Hercules, CA). Running temperature was 60 °C and 0.005 *M* H_2_SO_4_ was applied as mobile phase with a flow rate of 0.6 mL/min. Each product was calibrated by its standard solutions with five different concentrations (e.g. 1, 2, 3, 4, 5 g/L). Similarly, sugars from the composition analysis of biomass were analyzed by the HPLC and Hi-Plex Pb column. The mobile phase was de-ionized water at 0.5 mL min^−1^. Temperatures of the detector and the column oven were 55 °C and 70 °C, respectively. Concentrations of the monomeric sugar standards were 0.05, 0.1, 0.5, 1, 1.5 and 2 g L^−1^. All the standard calibration curves for HPLC analysis obtained with R^2^ > 0.998 were used for quantitative calculation.

Microbial mass (DCW, g/L) was determined through measuring the absorbance of broth at 650 nm (OD_650_) by the UV-visible spectrophotometer. One unit of optical density (OD_650_) was estimated to be equal to 0.36 g/L DCW (y = 0.2835x + 0.0846, R^2^ = 0.9974, Figure S2). Besides cross-checked by HPLC, glucose in liquid samples after fermentation was mainly quickly determined using a biosensor (SBA-40, Institute of Biology, Shandong Academy of Sciences, Jinan) calibrated with glucose standard solution (100 mg/dL). TRS in the hydrolysates after two-step hydrolysis or fermentation was determined by the UV spectrophotometer using dinitrosalicylic acid (DNS) method^43^ calibrated with five different concentrations (0, 0.2, 0.4, 0.6, 0.8 and 1.0 g/L) with R^2^ > 0.9999.

## Additional Information

**How to cite this article**: Jiang, D. *et al.* Biohydrogen Production from Hydrolysates of Selected Tropical Biomass Wastes with *Clostridium Butyricum.* *Sci. Rep.* **6**, 27205; doi: 10.1038/srep27205 (2016).

## Supplementary Material

Supplementary Data

## Figures and Tables

**Table 1:** Variables and their levels employed in Plackett-Burman design.

Factor (g/L)	Variables	Levels^a^	
		−1	1
Glucose	X_1_	15	25
Yeast extract	X_2_	3.5	6.5
Tryptone	X_3_	3	5
K_2_HPO_4_	X_4_	2	4
KH_2_PO_4_	X_5_	2	4
L-Cysteine	X_6_	0.05	0.15
MgSO_4_·7H_2_O	X_7_	0.0	0.1
FeSO_4_·7H_2_O	X_8_	0.2	0.4

^a^x_1_ = (X_1_ − 20)/5; x_2_ = (X_2_ − 5)/1.5; x_3_ = (X_3_ − 4)/1; x_4_ = (X_4_ − 3)/1; x_5_ = (X_5_ − 3)/1; x_6_ = (X_6_ − 0.1)/0.05; x_7_ = (X_7_ − 0.05)/0.05; x_8_ = (X_8_ − 0.3)/0.1.

**Table 2:** Plackett-Burman design variables (in code levels) with H_2_ yield as response for 24 h bottle fermentation at 35 °C with 130 rpm shaking.

Run	Variable level								H_2_ yield (mol/mol)^a^
	X_1_	X_2_	X_3_	X_4_	X_5_	X_6_	X_7_	X_8_	
1	−1	−1	−1	1	−1	−1	1	−1	1.39 ± 0.08
2	−1	1	−1	−1	1	−1	1	1	1.50 ± 0.02
3	−1	−1	1	−1	−1	1	1	1	1.41 ± 0.07
4	−1	−1	−1	1	−1	−1	1	−1	1.36 ± 0.05
5	−1	1	1	1	−1	−1	−1	1	1.44 ± 0.03
6	1	1	−1	−1	−1	1	−1	−1	1.30 ± 0.09
7	−1	1	−1	1	1	1	−1	−1	1.41 ± 0.05
8	1	−1	1	1	1	−1	−1	−1	1.28 ± 0.05
9	1	−1	−1	−1	1	−1	−1	1	1.32 ± 0.05
10	1	1	1	1	1	1	1	1	1.35 ± 0.01
11	1	−1	−1	1	−1	1	1	1	1.23 ± 0.04
12	1	1	1	−1	−1	−1	1	−1	1.34 ± 0.05

^a^Values were given by mean  ±  standard deviation (n = 3).

**Table 3 t3:** Effects and statistical analysis of variables^a^.

Variable	Coefficient	Std. Error	*t*-Value	*p*-Value
Intercept	1.3644	0.0054	251.54	<0.0001^b^
X_1_	−0.0611	0.0054	−11.27	0.0015^b^
X_2_	0.0256	0.0054	4.71	0.0181^b^
X_3_	0.0078	0.0053	1.45	0.2439
X_4_	−0.0161	0.0053	−3.00	0.0579
X_5_	0.0167	0.0056	2.96	0.0593
X_6_	−0.0122	0.0053	−2.27	0.1077
X_7_	0.0067	0.0055	1.22	0.3112
X_8_	0.0083	0.0055	1.52	0.2260

^a^R^2^ = 0.9858, R^2^ (Adj) = 0.9478. ^b^Statistical signification at 95% of confidence level (*p* < 0.05).

**Table 4:** Experimental results along the path of the steepest ascent for 24 h bottle fermentation at 35 ^o^C with 130 rpm shaking.

Test	Glucose (g/L)	Yeast extract (g/L)	H_2_ yield (mol/mol) a
1	20	5.0	1.62 ± 0.12
2	18	5.5	1.63 ± 0.03
3	16	6.0	1.78 ± 0.02
4	14	6.5	1.51 ± 0.03
5	12	7.0	1.33 ± 0.02
6	10	7.5	1.31 ± 0.28
7	8	8.0	1.20 ± 0.01

^a^Values were given by mean ± standard deviation (n = 3).

**Table 5:** Levels of the factors, experimental design and the results of the central composite design.

Run	Coded variable level		Real variable level		H_2_ yield (mol/mol-TRS)^a^	
	X_1_	X_2_	X_1_	X_2_	Observed	Predicted
1	1	−1	17	5.5	1.62 ± 0.03	1.61
2	0	0	16	6.0	2.02 ± 0.05	2.00
3	−1.41	0	14.59	6.0	1.84 ± 0.01	1.86
4	−1	−1	15	5.5	1.79 ± 0.08	1.78
5	0	0	16	6.0	1.99 ± 0.02	2.00
6	0	0	16	6.0	1.98 ± 0.01	2.00
7	0	0	16	6.0	2.01 ± 0.02	2.00
8	0	−1.41	16	5.29	1.64 ± 0.03	1.66
9	0	1.41	16	6.71	1.71 ± 0.07	1.72
10	−1	1	15	6.5	1.85 ± 0.09	1.83
11	0	0	16	6.0	2.01 ± 0.02	2.00
12	1.41	0	17.41	6.0	1.61 ± 0.02	1.61
13	1	1	16	6.5	1.63 ± 0.04	1.63

^a^Values were given by mean ± standard deviation (n = 3).

**Table 6:** Significance test of regression coefficient^a^.

Variable	Coefficient	Std. Error	*t*-Value	*p*-Value
Intercept	2.002	0.0088	227.04	<0.0001^b^
X_1_	−0.0894	0.0070	−12.83	<0.0001^b^
X_2_	0.0211	0.0070	3.03	0.0191^b^
X_1_X_2_	−0.0125	0.0098	−1.27	0.2454
X_1_X_1_	−0.1329	0.0075	−17.77	<0.0001^b^
X_2_X_2_	−0.1579	0.0075	−21.12	<0.0001^b^

^a^R^2^ = 0.9918, R^2^ (Adj) = 0.986. ^b^Statistical signification at 95% of confidence level (*p* < 0.05).

**Table 7:** Analysis of variance and canonical analysis for the parameters of RSM.

Source	Degree of freedom	Sum of square	Mean square	*F*-value	Probability > *F*
Model	5	0.330755	0.066151	170.1574	<0.0001
Error	7	0.002722	0.000389		
C. total	12	0.333477			
Lack of fit	3	0.001641	0.000547	2.0264	0.2528
Pure error	4	0.001088	0.000270		
Canonical analysis					
	X_1_	X_2_	Predicted H_2_ yield: 2.02 (mol/mol)		
Eigen value	−0.3402	0.0804			
Actual value	15.6598	6.0402			

**Table 8:** Structural carbohydrates and lignin in bagasse and *Jatropha* hulls^a^.

Biomass	Glucan (%)	Xylan (%)	Galactan (%)	Arabinan (%)	Mannan (%)	Lignin (%)	Ash (%)	Extractives (%)
Bagasse	42.05 ± 0.62	19.79 ± 2.81	2.47 ± 0.29	1.79 ± 0.02	1.12 ± 0.05	20.82 ± 0.12	1.98 ± 0.25	9.29 ± 0.01
*Jatropha* hulls	36.95 ± 1.35	9.78 ± 1.35	0.82 ± 0.03	0.30 ± 0.00	2.47 ± 0.53	27.9 ± 0.1	1.19 ± 0.19	18.61 ± 0.03

^a^All data were given by mean ± standard deviation (n = 3).

**Table 9:** Comparisons of product yields from glucose, hydrolysates of bagasse and *Jatropha* hulls for H_2_ production for 24 h fermentation at 35 °C with 130 rpm stirring/shaking in both reactors^a^.

Substrate	Reactor	H_2_ yield (mol/mol)	Acetic acid (AC) (g/L)	Butyric acid (BC) (g/L)	2-propanol (g/L)	AC/BC Ratio	Gas volume (mL)	Composition (%): H_2_	CO_2_
Glucose	Fermenter	2.15 ± 0.03	3.87 ± 0.12	3.35 ± 0.09	0.84 ± 0.01	1.15	8120 ± 37.98	64.34 ± 0.47	35.66 ± 0.40
	Bottle	2.03 ± 0.02	3.14 ± 0.06	2.87 ± 0.04	1.27 ± 0.02	1.09	233 ± 3.79	52.69 ± 0.14	47.31 ± 0.81
Bagasse hydrolysate	Fermenter	2.06 ± 0.02	3.46 ± 0.09	3.34 ± 0.07	1.36 ± 0.03	1.03	8532 ± 33.51	53.92 ± 0.61	46.08 ± 0.09
	Bottle	1.99 ± 0.03	2.28 ± 0.03	2.47 ± 0.02	1.24 ± 0.03	0.9	230 ± 5.69	50.16 ± 0.09	49.84 ± 0.04
*Jatropha* hulls hydrolysate	Fermenter	1.95 ± 0.06	2.37 ± 0.01	3.54 ± 0.03	1.15 ± 0.01	0.67	8767 ± 88.77	49.70 ± 0.49	50.29 ± 0.20
	Bottle	1.89 ± 0.02	1.97 ± 0.01	3.43 ± 0.03	1.20 ± 0.01	0.57	195 ± 1.49	56.62 ± 1.30	43.38 ± 0.24

^a^All data were given by mean ± standard deviation (n = 3).

**Table 10:** Comparison of biohydrogen yield in this study with reported data.

Raw biomass	Carbon resource	Microorganism	Reducing Sugar (g/L)	H_2_ yield (mol/mol)
Bagasse	Glucose Xylose	*C. butyricum*	20	1.73^18^
Bagasse	Glucose Xylose	*Clostridium sp.*	10	0.8^19^
Starch	Hexose	*C. butyricum*	5	2.0^20^
Glucose	Glucose	*C. butyricum* ATCC19398	3	1.8^37^
Glucose	Glucose	*C. butyricum*	2.5	1.4–2.3^38^
Glucose	Glucose	*Clostridium sp.*	20	0.85^36^
Glucose	Glucose	*C. butyricum*	15.64	2.15^a^
Bagasse	Glucose Xylose	*C. butyricum*	15.64	2.06^a^
*Jatropha* hulls	Glucose Xylose	*C. butyricum*	15.64	1.95^a^

^a^Data in this study in 3L fermenter.
