# Supplementary material for: Biohydrogen Production from Hydrolysates of Selected Tropical Biomass Wastes with Clostridium Butyricum
Source: Sci Rep. 2016 Jun 2;6:27205. doi: 10.1038/srep27205 (PMC4890049; doi:10.1038/srep27205)
Supplement: Supplementary Data [file srep27205-s1.pdf]

Supplementary Materials

Biohydrogen Production from Hydrolysates of Selected Tropical Biomass Wastes with *Clostridium Butyricum*

Dan Jiang[1,3], Zhen Fang*[2,3,1], Siew-xian Chin[3,4], Xiao-fei Tian[3,5], Tong-chao Su[1,3]

[1] University of Science and Technology of China, School of Life Science, 443 Huangshan Road, Hefei, Anhui Province 230022, China

[2] Biomass Group, College of Engineering, Nanjing Agricultural University, 40 Dianjiangtai Road, Nanjing, Jiangsu 210031, China

[3] Chinese Academy of Sciences, Biomass Group, Key Laboratory of Tropical Plant Resources and Sustainable Use, Xishuangbanna Tropical Botanical Garden, 88 Xuefulu, Kunming, Yunnan Province 650223, China

[4] Department of Chemical and Process Engineering, Faculty of Engineering and Built Environment, Universiti Kebangsaan Malaysia, 43600 UKM, Bangi, Selangor, Malaysia

[5] Department of Chemical Engineering, University of New Brunswick, 3 Bailey Dr, Fredericton, New Brunswick, E3B 5A3, Canada

*Author for correspondence, zhen.fang@mail.mcgill.ca

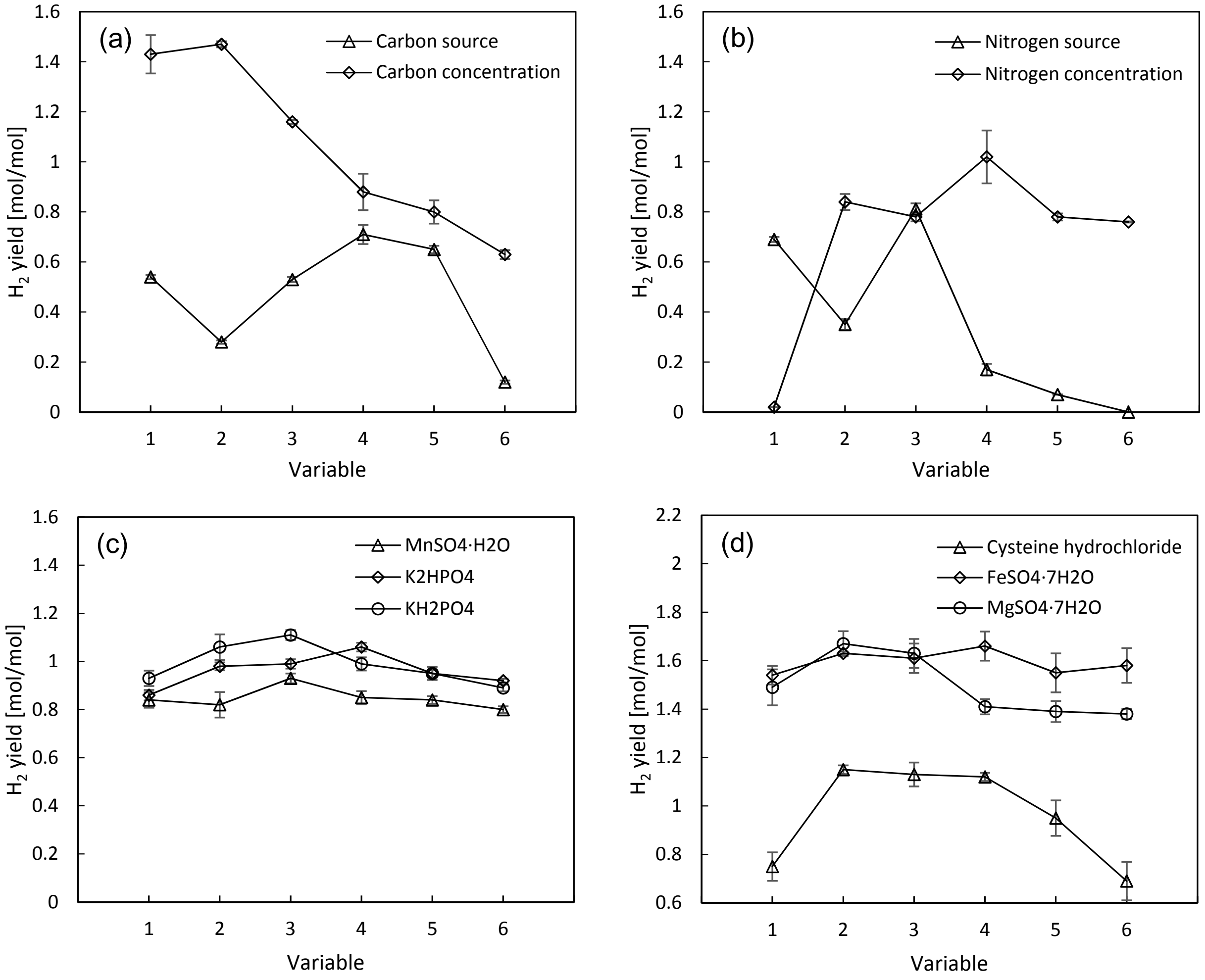

Figure S1. H_2 yield versus variable under the initial medium condition for 24 h bottle fermentation at 35 ^oC and 130 rpm shaking:
(a) carbon sources (No.1-6: Xylose, Galactose, Fructose, Glucose, Mannose, Glycerol; 20g/L), carbon concentrations (No.1-6: 10, 15, 20, 25, 30, 35 g/L); (b) nitrogen sources (No.1-6: tryptone, peptone, yeast extract, urea, $(NH_4)_2SO_4$, $NH_3 \cdot H_2O$; 10 g/L), nitrogen concentrations (No.1-6: 0, 1, 3, 5, 7, 9 g/L); (c) concentrations of $MnSO_4 \cdot 7H_2O$ (No.1-6: 0, 0.05, 0.1, 0.15, 0.2, 0.25 g/L), concentrations of K_2HPO_4 and KH_2PO_4 (No.1-6: 0, 1, 3, 5, 7, 9 g/L); (d) concentrations of cysteine hydrochloride and $MgSO_4 \cdot 7H_2O$ (No.1-6: 0, 0.05, 0.1, 0.15, 0.2, 0.25 g/L), concentrations of $FeSO_4 \cdot 7H_2O$ (No.1-6: 0, 0.1, 0.2, 0.3, 0.4, 0.5 g/L).

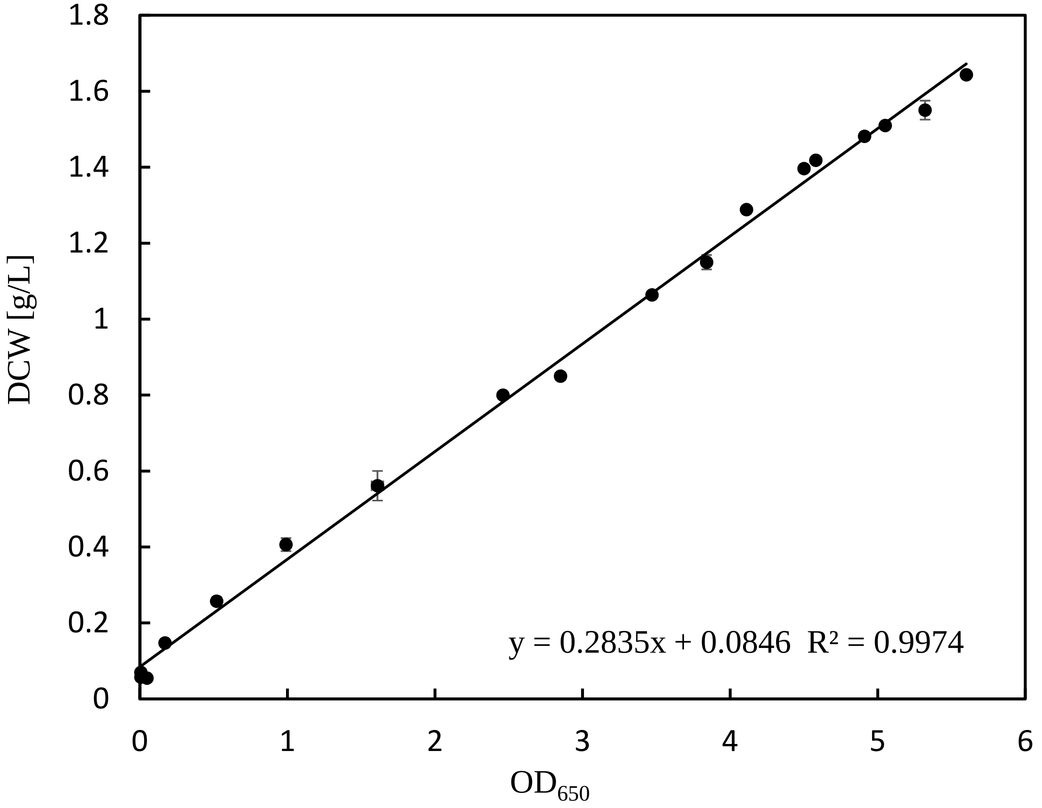

Figure S2. Regression curve between dry cell weight (DCW) and optical density (OD_{650}).

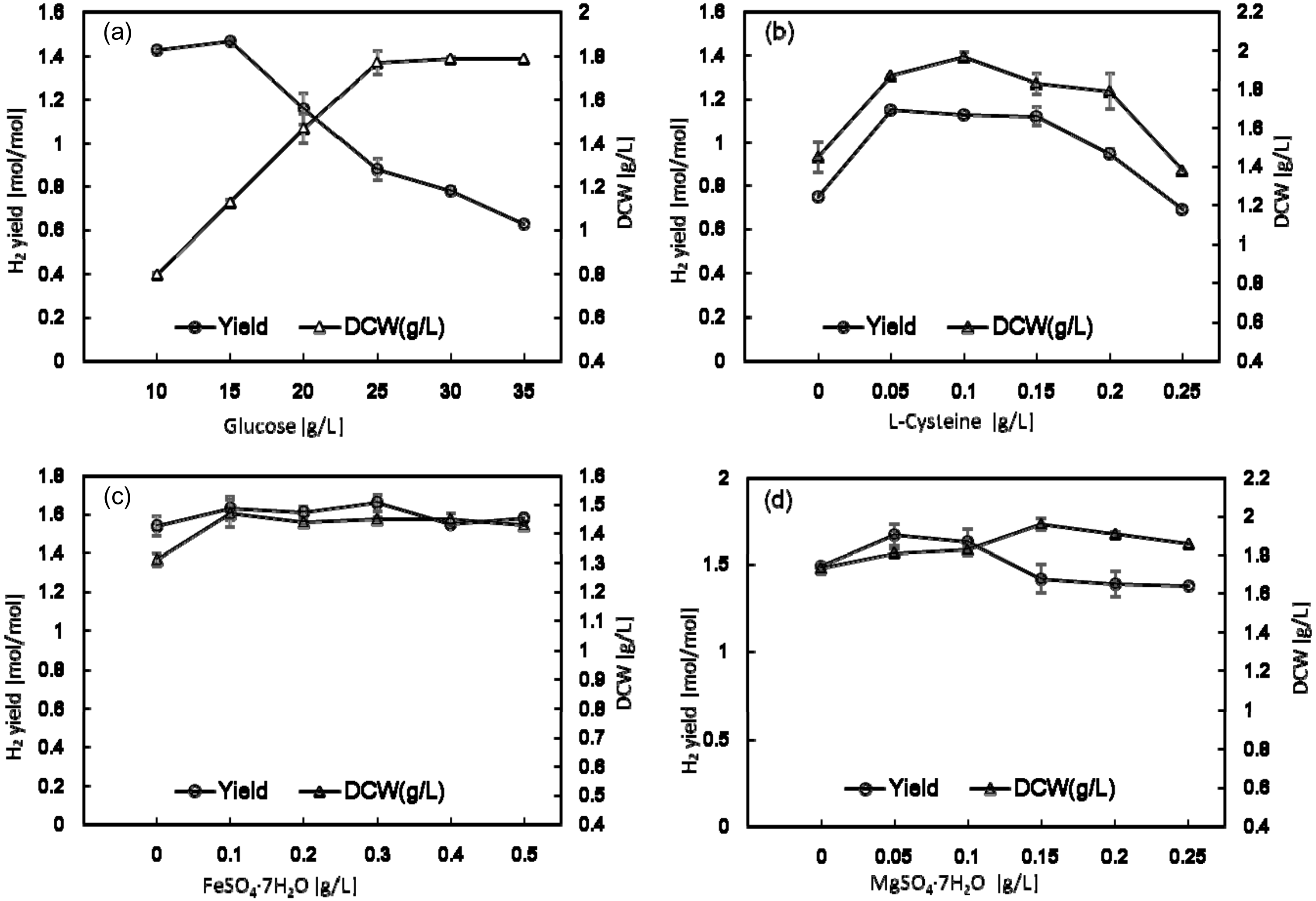

Figure S3. H_2 yield and dry cell weight (DCW) versus different concentration of (a) glucose, (b) L-cysteine, (c) $FeSO_4·7H_2O$, and (d) $MgSO_4·7H_2O$ (24 h bottle fermentation at 35 °C and 130 rpm shaking).
